# Anterior C2 Odontoid Fixation Using Cranial Brainlab Navigation: A Novel Approach and Case Report

**DOI:** 10.7759/cureus.73572

**Published:** 2024-11-13

**Authors:** Muhammad Mohsin Khan, Noman Shah, Omar M Shihadeh, Abdullah Illeyyan, Alaaeldin ali salih Ahmed, Amr Rachid El Mohamad, Thaha Muhammed, Abdulnasser Thabet, Sirajeddin Belkhair

**Affiliations:** 1 Clinical Research, University of Dresden, Dresden, DEU; 2 Neurosurgery, Hamad General Hospital, Doha, QAT; 3 Neurological Surgery, Hamad General Hospital, Doha, QAT; 4 Service Engineering, Sedeer Medical, Doha, QAT; 5 Clinical Academic Sciences, College of Medicine, Qatar University, Doha, QAT; 6 Neurological Sciences, Weill Cornell Medicine-Qatar, Doha, QAT

**Keywords:** cervical spine surgery, cranial brainlab navigation, ct-registration, mayfield skull clamp, odontoid screw fixation, type ii odontoid fractures

## Abstract

Instrumentation of the cervical spine particularly at the higher cervical levels like C2 presents unique challenges mainly because of their complex anatomy and proximity to neurovascular structures. The goal of the article is to demonstrate that using navigation technologies in inserting anterior odontoid screws can enhance the precision and safety of surgery. We describe a novel approach for anterior C2 odontoid fixation using a three-pin radiolucent Mayfield clamp with intra-operative CT registration and cranial brainlab navigation.

A 28-year-old male patient who had a type II odontoid fracture was treated by this innovative technique using a radiolucent Mayfield clamp and cranial Brainlab navigation system to increase the precision of screw placement in the cervical spine and reduce the usage of X-ray during the procedure. The application of this technique results in the appropriate placement of the odontoid screw with limited neck exposure and less operative time.

This novel surgical technique represents a significant advancement in the surgical treatment of patients with type II odontoid fractures by offering enhanced precision and safety and reduced surgical trauma. Its successful application in this challenging cervical spine surgery proves its potential in the anterior approach of cervical spine surgery.

## Introduction

Since the late 90s, there has been an evolution of new technologies to help in spinal surgery that is notably navigation-guided and minimally invasive systems. While starting with basic fluoroscopic guidance, the field has advanced to incorporate intra-operative computed tomography (iCT)-based navigation systems [1,2]. These computer-assisted navigation tools have revolutionized spinal surgeries by elucidating complex spinal anatomy in real time thus enhancing the precision of surgical procedures minimizing radiation exposure reducing the duration of surgeries and decreasing complication rates [3].

Intra-operative navigation has been extensively used in thoracolumbar spine surgeries and recently gained popularity in cervical spine procedures, especially in the area of craniocervical junction [4]. Cervical spine surgeries have distinct challenges due to the complex nature of the area, particularly at higher cervical levels due to proximity to the spinal cord and other vital structures notably the vertebral artery. Inserting pedicle screws in the lower cervical spine can be especially challenging with standard intra-operative x-ray. So the integration of intra-operative 3D navigation in cervical spine surgeries has significant advantages.

Odontoid fractures

Axis vertebra fractures that include type II odontoid fractures as described by Anderson and D’Alonso are the most common cervical spine fractures, and they account for 10%-20% of these fractures [5]. Type II odontoid fractures occur at the base of the odontoid process and are typically unstable if the transverse ligament is ruptured, often requiring surgical intervention for healing.

There is an ongoing debate regarding the most appropriate method of fixation for these fractures. However, the prevailing approach is posterior C1-C2 instrumentation to maintain the stability of the atlantoaxial complex and realign the structure to promote healing; several surgical techniques are available for treating unstable type II odontoid fractures [6]. The most common approach is Harms-type fusion which involves placing C-1 lateral mass and C-2 pars or pedicle screws, connected by rods [7]. The alternative common approach is anterior odontoid screw placement which is most suitable for fractures with a specific anterosuperior to posteroinferior orientation, provides direct fixation across the fracture site and preserves normal C1-2 rotational motion, which is often lost in posterior C1-2 fusion [8].

## Case presentation

**Figure 1:**
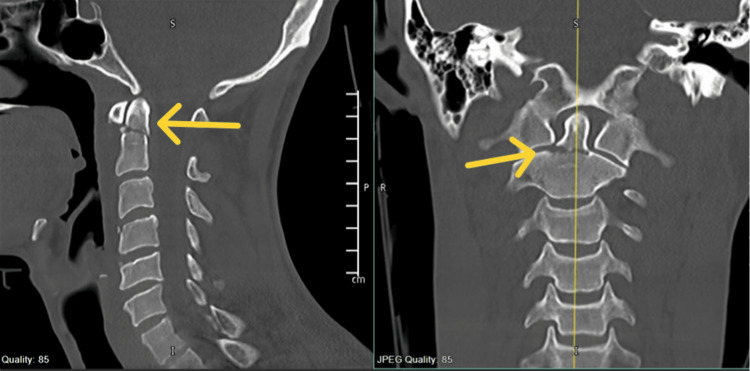
Pre-operative image showing sagittal and coronal views of type II odontoid fracture.

A 28-year-old male patient with no previous medical or surgical history presented after a fall from a height of eight meters while working on a crane. Following the incident, he was complaining of pain in the neck and thoracic (mid-back) regions, CT of the spine showed multiple cervical and thoracic spinal fractures including type II odontoid fracture. The patient was fully intact during the neurological examination (Figure 1).

Surgical technique

**Figure 2:**
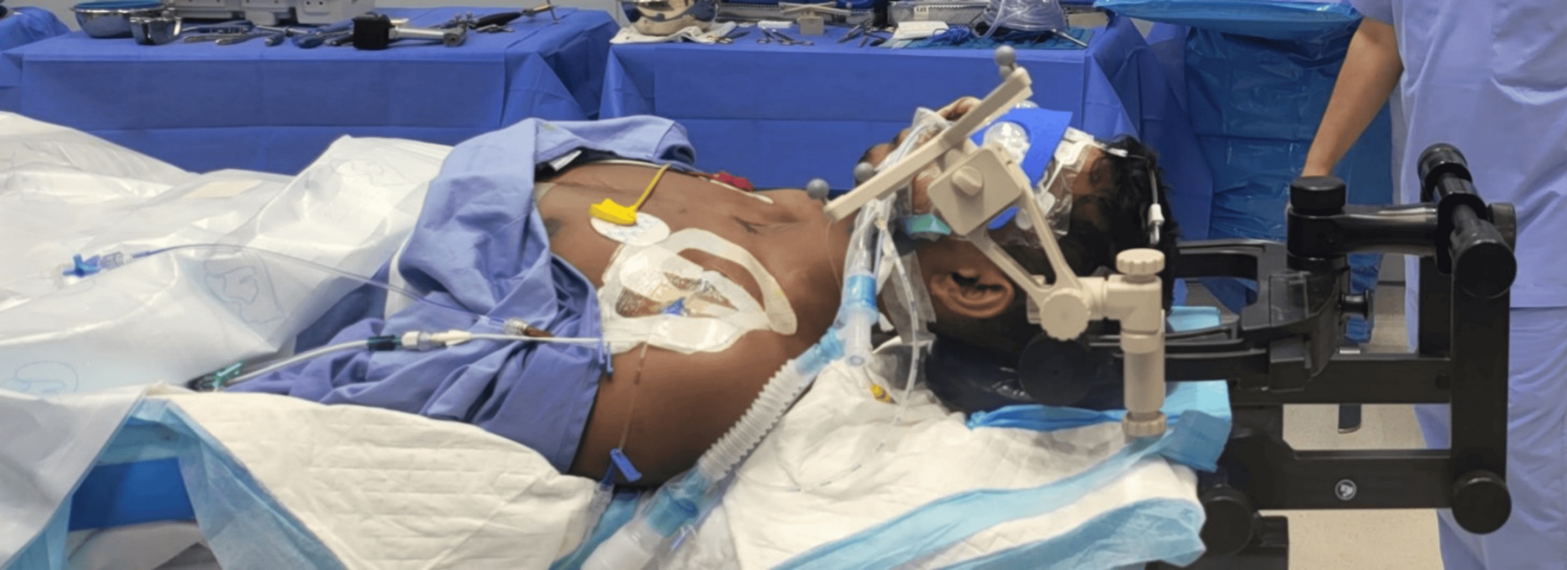
Intra-operative image showing the lateral view of the radiolucent Mayfield clamp attached to the patient's skull, with the application of a 4-star tripod for cranial Brainlab navigation.

**Figure 3:**
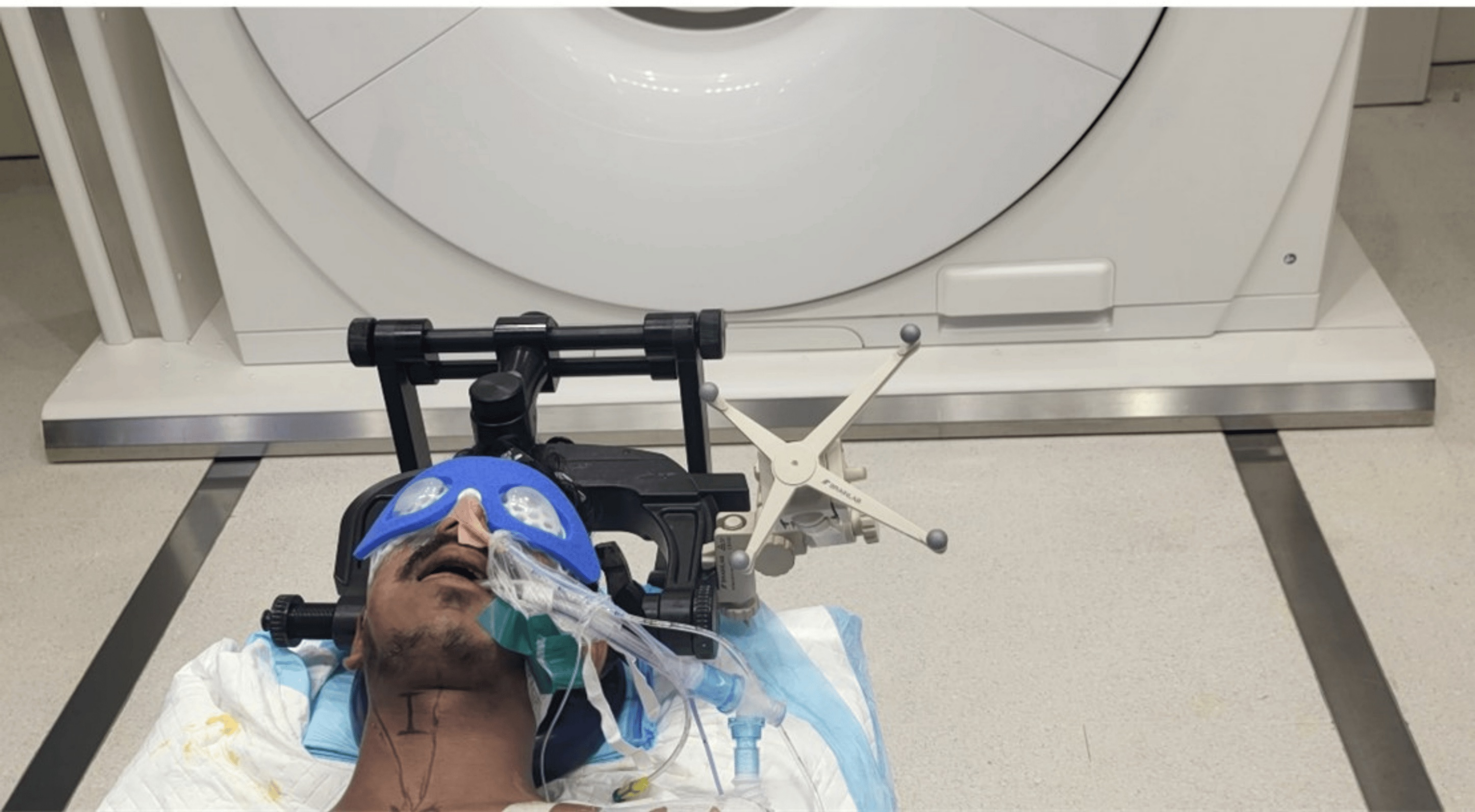
Intra-operative image showing the anterior view of the radiolucent Mayfield clamp attached to the patient's skull, with the application of the 4-star tripod for cranial Brainlab navigation.

**Figure 4:**
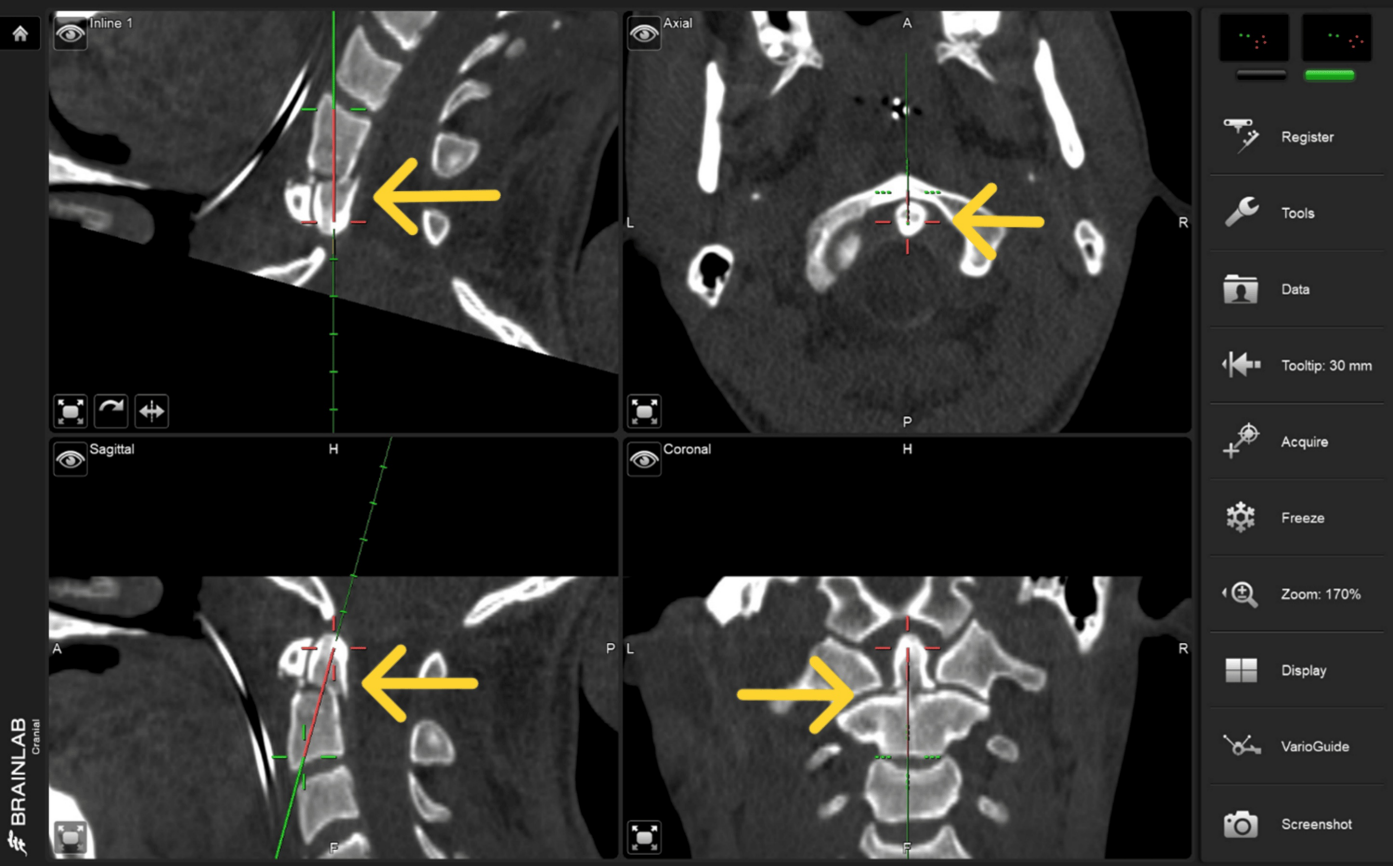
Pre-operative sagittal cervical spine image showing reduction of the dens after intubation to maintain proper alignment.

After performing fiber optic intubation, a radiolucent three-pin Mayfield skull clamp is attached. This clamp's bilateral pins are positioned two fingerbreadths above the ear pinna and aligned with the tragus. The brain lab radiolucent cranial navigation frame is connected to the Mayfield using a Mayfield adapter (Figures 2, 3). The Mayfield adapter is then utilized to extend the patient's head, facilitating the correct angle for odontoid screw insertion. Additionally, the head's crown is slightly bent forward to adjust the misaligned odontoid tip (Figure 4). Such positioning was vital to achieve the correct angle for odontoid screw placement.

As part of the instrument-guided surgical navigation technique, we have used the Brainlab navigation system. The patient setup for the navigation was done with the radiolucent cranial reference array (4-star tripod) attached to the radiolucent Mayfield fixed on the patient. This setup is selected for performing the Automatic image registration with the intraoperative CT. It’s a single-step registration process while doing the intraoperative CT and sending the CT image to the Brainlab curve navigation system to complete the registration. We then confirmed the accuracy of the registration by using the tip of the nose and incisor teeth thyroid cartilage and other anatomical landmarks on the patient facial points.

Following the registration acceptance, the cranial navigation window was started on the navigation screen, giving us the Brainlab pre-calibrated pointer to navigate the patient's anatomy with the help of the CT image on the screen.

The patient is meticulously prepared for surgery, adhering to standard sterile protocols. To ensure sterility, a sterile passive frame is used in the procedure. An oblique longitudinal incision was marked on the right side of the patient's anterior neck, the level of the thyroid cartilage, generally corresponding to the C4 spinal region. We selected an oblique longitudinal skin incision to give easy access to the anterior inferior portion of C2.

The incision is carefully dissected down to the platysma muscle is subsequently split using Bovie electrocautery, then is gently dissected from the surrounding tissue at the upper part and retracted with a self-retaining retractor. The dissection then proceeded along the medial border of the sternocleidomastoid muscle, utilizing a surgical scissor. Handheld Cloward retractors were used to carefully move the carotid artery, jugular vein, and vagus nerve to the side. Concurrently, an assistant retracted the trachea and esophagus towards the center of the body. This careful retraction is crucial for providing clear access to the surgical site while avoiding injuring neurovascular structures. We made a small hole with the diamond drill in the body of C4 to make it a reference point to check the accuracy of navigation throughout the procedure.

We made minimal dissection of the anterior vertebral body of C2 then the longus colli muscles, situated along the anterior vertebral bodies, are carefully mobilized away from the spine using electrocautery. Special attention was paid to avoid disturbing the intervertebral disc spaces during this process. The navigation probe was used to identify C2-3 disc space. Using the Brainlab navigation probe the starting point and trajectory for the odontoid screw placement were confirmed. The diamond drill was used to create an entry point for the path for the screw, then navigated drill carved a midline channel in the upper part of the C-3 vertebrae. This channel is designed to house the head of the odontoid screw.

**Figure 5:**
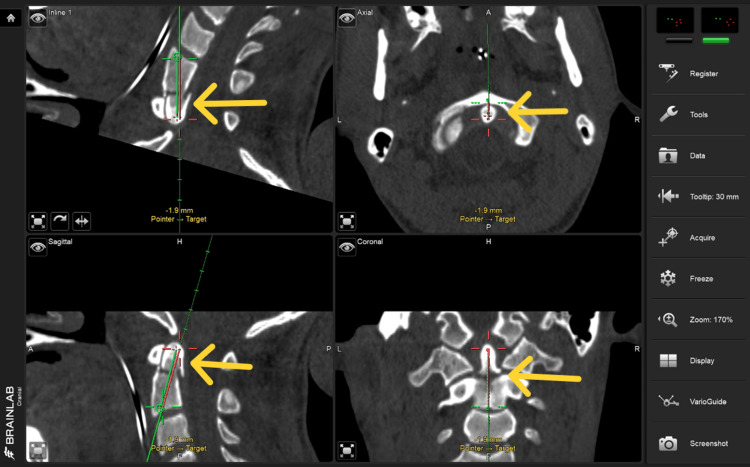
Intra-operative Brainlab navigation screen showing the trajectory of the calibrated navigation pointer that was used for placement of the k-wire.

The offset option available in the cranial navigation tools was utilized for the depth calculation and to plan the path of the screw placement (Figure 5).

**Figure 6:**
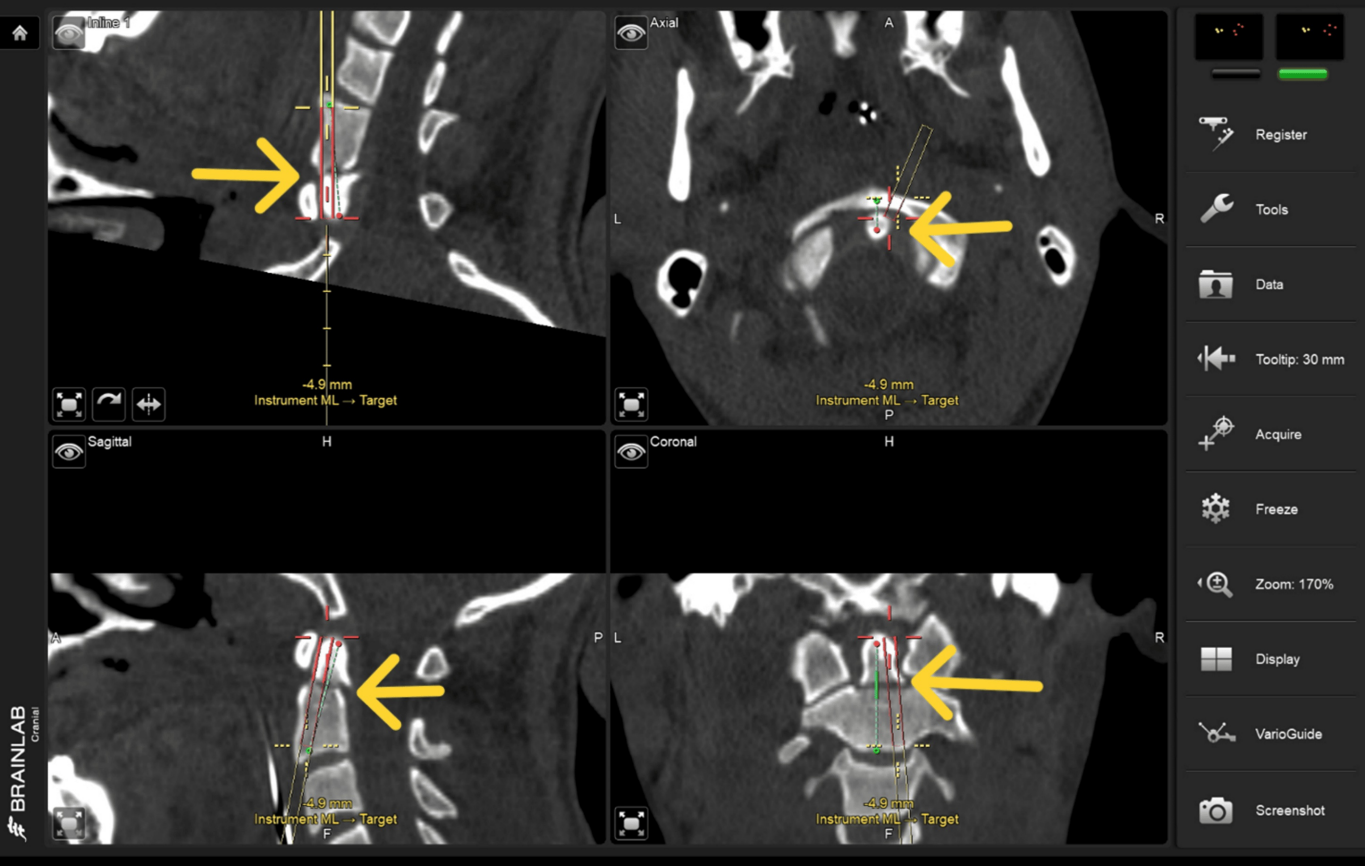
Intra-operative Brainlab navigation screen for trajectory of the drill that was calibrated through instrument adapter fixation (3-star medium size) on the drill guide and calibrated with the ICM4 (Instrument calibration matrix), this navigated drill was used to drill the trajectory for the final placement of the screw.

The k-wire was then placed using the trajectory determined by the brain lab-calibrated pointer. Then the position of the wire was checked with fluoroscopy. During the entry point and screw path identification the drill guide instrument was calibrated with the help of instrument adapter fixation (3-star medium size) on the drill guide and calibrated with the ICM4 (Instrument calibration matrix), the instrument was used for the navigation guidance after verifying the accuracy. This drill registered to the brain lab system was used to drill the trajectory (Figure 6), this navigated drill was used to make a trough on the anterior surface of the C-3 vertebral body to reach the anterior inferior part of C-2, aiming towards the odontoid process's tip. The accuracy was checked again with a hole made in the anterior c4 body, as an extra precaution, anterior-posterior (AP) and lateral fluoroscopic images were captured using the X-ray at 5 mm intervals. This step is crucial to verify in real time that the drill's actual position aligns with the intended trajectory.

While this fluoroscopy check was not necessary as the Brainlab navigation system accuracy was repeatedly checked before every step, we recommended it as an additional safety measure, rather than solely relying on the planned trajectory. Once the drilling was complete the drill was removed then a tap was used through the drilled portion length. The correct screw length is determined based on the measured distance from the lower part of C-2 to the odontoid process's tip which was 36mm measured using CT images generated intraoperatively by the brain lab. A lag screw was preferred for aligning a displaced fracture was then inserted. The insertion was done using a screw guide allowing the screw to naturally find its path. Throughout this process, AP and lateral radiographs are taken to confirm the screw's trajectory.

**Figure 7:**
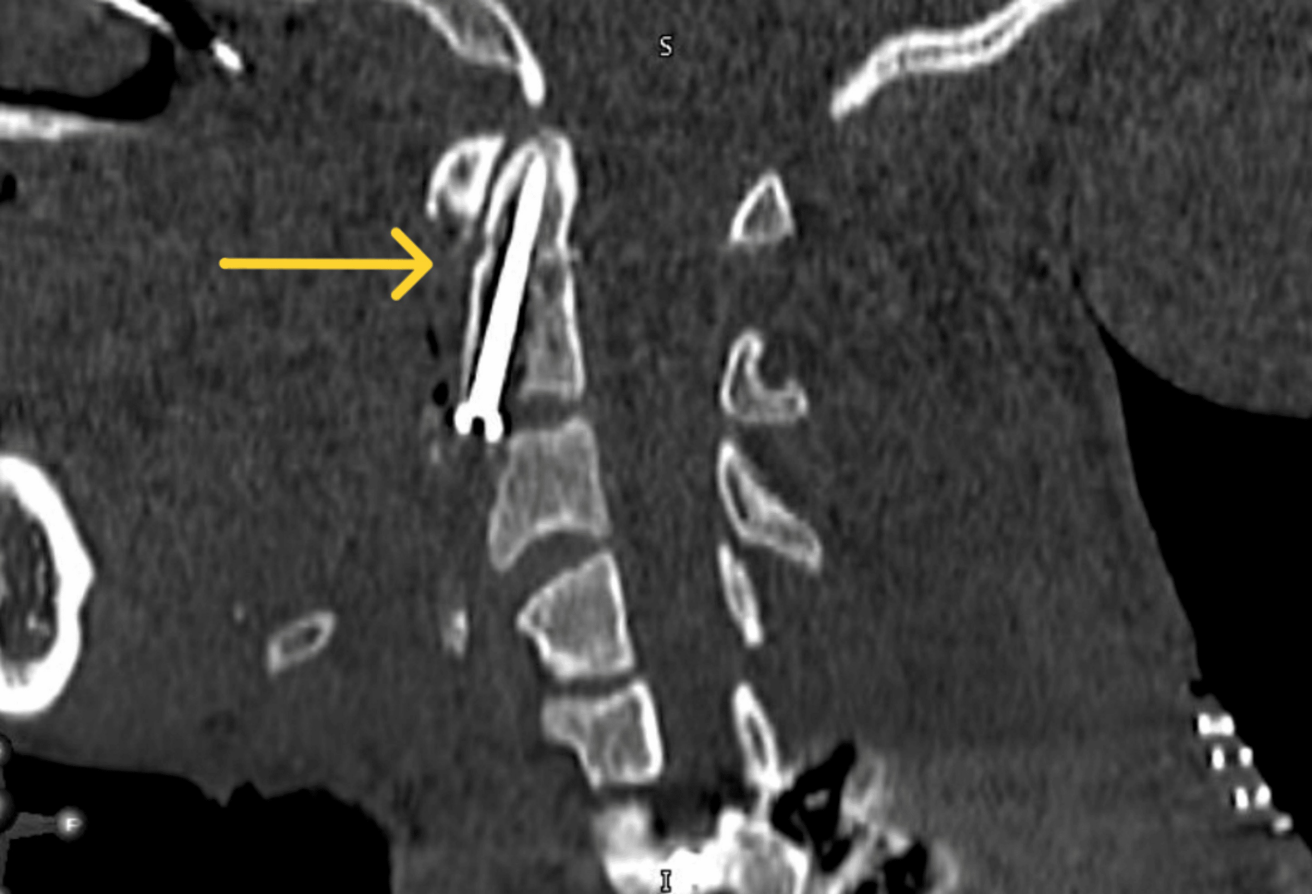
Intra-operative post-fixation sagittal cervical spine image showing the final placement of the screw.

**Figure 8:**
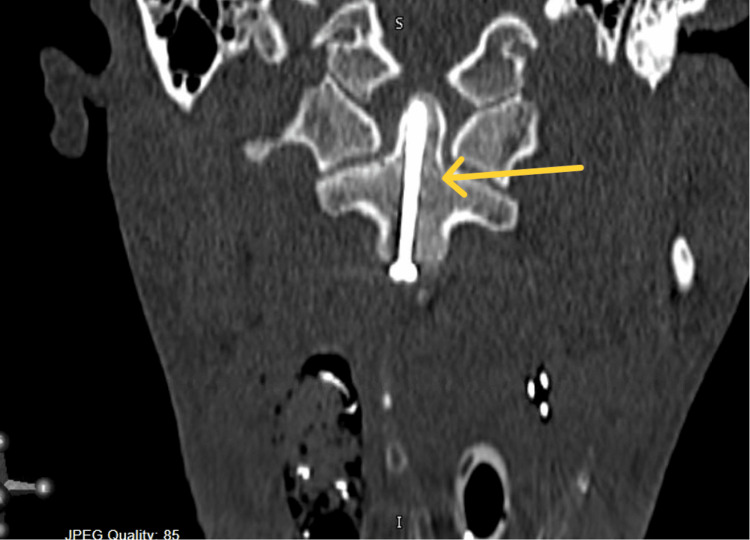
Intra-operative post-fixation axial cervical spine image showing the final placement of the screw.

Unlike the drilling phase where both navigation and fluoroscopy were employed screw insertion relied solely on fluoroscopy as the planned trajectory remains unchanged in real-time. The screw is positioned such that its head is aligned with the front surfaces of the C-2 and C-3 vertebral bodies. Following the removal of retractors, for the final verification, the intra-operative CT was performed to confirm the screw position (Figures 7, 8).

Upon achieving hemostasis, the surgical drain was inserted, and the platysma was closed with Vicryl 4/0 followed by closure of the subcutaneous layer by 3-0 Vicryl sutures which are placed in an interrupted fashion. The skin was sutured with 4-0 absorbable subcuticular suture, which is applied in a running subcuticular stitch pattern. Steri-strips were applied and the wound was securely sealed with a Tegaderm dressing.

## Discussion

Observations

Type II odontoid fracture is the most common type of odontoid fracture and usually requires surgical fixation to restore stability and promote healing. The head and neck maintain their full range of motion. Various techniques have been employed to stabilize these fractures, most commonly utilized surgical approaches are anterior odontoid screw placement and posterior cervical fixation of C1 and C2 through various surgical techniques. Both of these surgical approaches have been developed and refined over time [9].

Anterior odontoid screw placement is considered best for traumatic type II odontoid fractures in adults. The general surgical technique involves general endotracheal anesthesia and positioning the patient supine. Precise screw placement is crucial for optimal results, and real-time biplanar fluoroscopy helps to achieve accurate control during the operation. In this technique, a Kirschner wire needs to be placed to guide the screw to its position [10]. Followed by the placement of the guide and using the drill through that under fluoroscopy, in our case with the help of using a navigated drill, we achieved a high level of safety and confidence for using the drill and visualizing the real-time progress on the navigation screen.

The role of posterior cervical instrumented fusion in managing odontoid type II fractures especially in cases where there was significant displacement or instability of atlantoaxial joints. Techniques like the Harms method offer alternatives to anterior screw placement, especially in patients with challenging anatomical variations or alignment issues. These posterior approaches while more effective in causing bony fusion will result in a loss of normal C1-C2 rotatory motion.

Some surgeons might feel uncomfortable with inserting anterior odontoid screws as they might find it challenging to visualize and control the insertion of guide wire using an X-ray; in our case with the help of novel integration and a combination of technologies like intra-operative CT registration and Brainlab navigation for the surgical procedure has been a game-changer. These technologies not only enhance the precision of instrument placement but also minimize the invasiveness of the methods by providing real-time navigational guidance they ensure optimal alignment and placement of the odontoid screw, thereby improving overall surgical outcomes and patient safety and making the procedure easier for spine surgeons.

Advantages of the technique

In our case which is described here, it is essential to use a radiolucent Mayfield clamp to maintain the accuracy of registration and navigation using intra-operative CT images and cranial Brainlab navigation. To the best of our knowledge this technique has never been applied before in placing C2 anterior odontoid screw, several key advantages of this technique are as follows:

Enhanced Precision and Safety

The integration of the Brainlab navigation system with intraoperative CT imaging greatly enhances the precision of screw placement. This is especially crucial in the delicate and complex anatomy of the cervical spine where the margin for error is minimal. It eliminates the requirement of using a spinal reference array that is difficult to place in cervical spine surgery, especially the anterior approach. This integration described in this case can be utilized in the posterior approaches like lateral mass screws as in the traditional method of navigation, the references array can cause nuance by coming in the way of surgical equipment and causing hindrance and is impractical in the anterior cervical approaches, especially for C2 anterior odontoid screw fixation.

Reduced Surgical Intra-operative Time, Trauma and Complications

This technique is allowing real-time better visualization of patients' vertebrae and surgical instruments (drill and tape) and which will allow more accuracy in placing odontoid screws and minimizes the need for extensive tissue dissection and manipulation due to confidence given by the navigation and that will ultimately lead to reduced surgical time and decrease number of x-ray and fluoroscopy taken during the surgery that is of paramount importance in trauma and unstable patients, This can lead to a lower risk of complication from anesthesia and faster recovery times. It is worth mentioning that for our first case, the procedure was completed within 2h of incision time.

Improved Surgical Outcomes

The precise placement of the odontoid screw facilitated by this advanced navigation system ensures optimal alignment and stabilization of the fracture. This promotes effective healing and preserves the normal motion of the C1-C2 rotatory movement.

Versatility and Applicability

Although demonstrated in the case of a C2 odontoid fracture this technique's principles can be applied to other complex anterior cervical spinal surgeries expanding its potential benefits to a broader range of spinal pathologies.

Innovative Integration of Technology

The adaptation of cranial navigation tools for spinal surgery is a novel approach that might set a precedent for future surgical techniques where cross-disciplinary technologies can be employed to enhance surgical outcomes.

## Conclusions

Intraoperative CT and navigation with Brainlab represent a quantum leap in cervical spine surgery but, most importantly, in the management of difficult Type II odontoid fractures. New technology allows the accuracy of screw placement without reliance on conventional spinal reference arrays, with very limited reliance on fluoroscopy. Appropriate real-time guidance enhances surgical safety while limiting radiation exposure and operative time without sacrificing the important C1-C2 rotatory motion. It does this by ensuring that the recovery of the patients is much faster with fewer complications. Such procedures set new standards both in precision and efficiency.
